# Calcium Regulates Growth and Nutrient Absorption in Poplar Seedlings

**DOI:** 10.3389/fpls.2022.887098

**Published:** 2022-05-10

**Authors:** Xiaohang Weng, Hui Li, Chengshuai Ren, Yongbin Zhou, Wenxu Zhu, Songzhu Zhang, Liying Liu

**Affiliations:** ^1^College of Forestry, Shenyang Agricultural University, Shenyang, China; ^2^Research Station of Liaohe-River Plain Forest Ecosystem, Chinese Forest Ecosystem Research Network (CFERN), Shenyang Agricultural University, Shenyang, China; ^3^Institute of Modern Agricultural Research, Dalian University, Dalian, China

**Keywords:** poplar growth, calcium, photosynthetic characteristics, stress resistance, nutrients absorption

## Abstract

As a crucial element for plants, calcium (Ca) is involved in photosynthesis and nutrient absorption, and affects the growth of plants. Poplar is an important economic forest and shelter forest species in China. However, the optimum calcium concentration for its growth is still unclear. Herein, we investigated the growth, biomass, photosynthetic pigments, photosynthetic parameters and products, chlorophyll fluorescence parameters, water use efficiency (iWUE), and antioxidant enzyme activity of “Liao Hu NO.1” poplar (*P. simonii* × *P. euphratica*) seedlings at 0, 2.5, 5, 10, and 20 mmol·L^−1^ concentrations of Ca^2+^, and further studied the absorption, distribution, and utilization of nutrient elements (C, N, P, K, and Ca) in plants. We found that with increasing calcium gradient, plant height and diameter; root, stem, leaf, and total biomasses; net photosynthetic rate (Pn); stomatal conductance (Gs); intercellular carbon dioxide (Ci) level; transpiration rate (Tr); *Fv/Fm* ratio; *Fv/F0* ratio; chlorophyll-a; chlorophyll-b; soluble sugar and starch content; superoxide dismutase (SOD), catalase (CAT), and peroxidase (POD) levels; and long-term water use efficiency (iWUE) of poplar seedlings first increased and then decreased. These parameters attained maximum values when the calcium concentration was 5 mmol·L^−1^, which was significantly different from the other treatments (*P* < 0.05). Moreover, a suitable Ca^2+^ level promoted the absorption of C, N, P, K, and Ca by various organs of poplar seedlings. The absorption of C, N, P, and K increased first and then decreased with the increased calcium concentration, but the optimum calcium concentrations for the absorption of different elements by different organs were different, and the calcium concentration in leaves, stems, and roots increased gradually. Furthermore, the increase in exogenous calcium content led to a decreasing trend in the C/N ratio in different organs of poplar seedlings. C/P and N/P ratios showed different results in different parts, and only the N/P ratio in leaves showed a significant positive correlation with Ca^2+^ concentration. In conclusion, the results of this study indicate that 5 mmol·L^−1^ concentration of Ca^2+^ is the optimal level, as it increased growth by enhancing photosynthesis, stress resistance, and nutrient absorption.

## Introduction

Among all the nutrients required for plant growth and development, calcium is an essential element. On the one hand, calcium plays an important role in various physiological functions of plants, such as regulating plant growth and development as a ubiquitous second messenger and responding to various biological and abiotic stresses (Hochmal et al., 2015; Kudla et al., 2018). On the other hand, calcium is an important component of the cell wall and membranes, thus helping in maintaining the normal structure and function of cells and reducing or delaying damage to the cell membrane (Hocking et al., 2016). At the same time, calcium regulates the mitosis of plant cells by controlling the spatial and temporal distribution of calcium ions or their receptors (calmodulin), which is positively correlated with the consumption of organic carbon (Hepler, 1994; Zhang and Liu, 2021). Calcium also affects the ability of plants to absorb other elements, and there are antagonistic and synergistic effects between other elements (Jackson, 1967; Arif et al., 2016). Over the past several decades, despite the rich content of calcium in the soil, people have not paid attention to it. In recent years, with the deepening of calcium research, new physiological functions of calcium have been recognized, and research on the role of calcium in plant growth has attracted increasing attention. When plants encountered Ca deficiency, the chlorophyll fluorescence parameters of tomato, *Fm* and *Fv/Fm*, dropped below the control level, and superoxide dismutase activity was also reduced (Schmitz-Eiberger et al., 2002). Calcium deficiency depressed plant growth in peach, damaged cell membranes, and impaired chlorophyll and chlorophyll precursor biosynthesis (Aras et al., 2021). However, excessive absorption of calcium ions can induce the closure of leaf stomata, thereby reducing photosynthesis and resulting in slow growth (Blatt, 2000). In addition, excessive free Ca^2+^ in the cytoplasm can precipitate ${\text{PO}}_{4}^{3-}$ ions, interfere with physiological processes related to phosphorus metabolism, inhibit respiration, and affect plant growth by hindering normal signal transduction (Hirschi, 2004). Studies have shown that the absorption and utilization of magnesium in tea plants are hindered by excessive calcium treatment, and the utilization efficiency of light energy in leaves is reduced, thus affecting the growth of new shoots and inhibiting the growth of roots in tea plants (Wang et al., 2010; Liang et al., 2021b). In addition, in a high-calcium environment, the metabolism of photosynthetic pigment of golden flower tea is disturbed, and the synthesis of chlorophyll is inhibited, which leads to a decrease in the light capture ability and photosynthetic activity of leaves and a decrease in photosynthetic capacity, thus eventually inhibiting plant growth (Chai et al., 2021). Meanwhile, with the increase in the calcium supply level, the content of nitrogen, phosphorus, potassium, zinc, and manganese in tobacco plants increased first and then decreased, while the content of calcium increased gradually (Jie et al., 2005; Chang et al., 2020). Studies have shown that both Ca deficiency and excessive Ca inhibit N absorption and utilization, and the adverse effects of Ca deficiency on seedling growth and N metabolism are greater than those associated with excessive Ca^2+^ supply (Xing et al., 2021). An appropriate calcium content could significantly promote nutrient absorption and accumulation under salt stress conditions and improve the distribution ratio of nitrogen, phosphorus, and potassium in mature pods of peanut (Shi et al., 2018). Although there is a correlation between calcium and plant photosynthetic characteristics and the absorption of other nutrient elements, calcium deficiency and calcium overload affect plant photosynthetic characteristics and the absorption of other nutrient elements, thus inhibiting plant growth. However, it is not clear whether excessive calcium inhibits the growth of shelterbelt tree species by affecting the photosynthetic characteristics and the absorption of other nutrient elements.

Poplar has the fastest growth rate among all the trees inhabiting temperate climates. Poplar's physiological traits are wide adaptability, strong survivability, and high biomass yield, and poplar can absorb excess carbon (Cândea-Crăciun et al., 2019; Henner et al., 2020). Poplar has been planted as a short rotation coppice and as an economic forest and ecological shelterbelt in large Chinese areas for wind speed reduction, sand fixation, and soil and water conservation (Zhou et al., 2013; Yan et al., 2017; Ahmed et al., 2020). However, due to the one-sided pursuit of economic value in early planting and the lack of proper planning, many problems occurred in these large-scale poplar plantations, such as top bud death, premature tree decay and death, soil and ecosystem degradation, and so on (Zhou et al., 2020). In recent years, the development of science and society has led to global warming, reduced soil moisture content, and resulted in other problems, which have further accelerated the decline of poplar plantations, resulting in the overall reduction in their resistance to stress, a reduction in protection efficiency, and thus the phenomenon of the absence of forest networks (Dhillon et al., 2017; Feng et al., 2018). At present, studies on poplar plantation restoration and nutrient absorption mainly focus on N addition (Jiang et al., 2021), mitigation of drought stress (Li et al., 2021), and root pruning (Jing et al., 2018), and less focus is placed on calcium. Therefore, it is necessary to explore the effect of calcium on nutrient absorption in poplar plants and promote nutrient absorption, utilization, and transformation in poplar plantations to improve stress resistance and productivity.

To meet the needs of a growing world for socioeconomic development and environmental protection, it is necessary to study the effects of calcium on the growth, physiological characteristics, and absorption of elements by various organs of poplar plants. The test material “Liao Hu NO.1” poplar is a hybrid of natural pollination “*P. simonii* × *P. euphratica*” in the F1 generation. It has the characteristics of fast growth, salt-alkali tolerance, easy reproduction, and cold resistance (Wang and Peng, 2015). A greenhouse incubation experiment was carried out with treatments including five calcium concentration gradients (0, 2.5, 5, 10, and 20 mmol·L^−1^). The objectives of this study were as follows: (1) to determine the optimum calcium concentration for poplar seedling growth; (2) to investigate mineral element resorption and utilization by the organs of poplar seedlings in the presence of five concentrations of calcium; and (3) to determine the C, N, and P concentrations and ecological stoichiometry of the different organs under different calcium levels.

## Materials and Methods

### Cultivation of Poplar Seedlings

This experiment was carried out in the greenhouse of Shenyang Agricultural University from April 2019 to July 2019. The experimental materials were seedlings of the annual poplar *P. simonii* × *P. euphratica*, which is a fast-growing, salt-tolerant species called “Liao Hu NO.1” poplar in China. Six poplar seedlings were planted into one pot with dimensions of 32 cm length × 18 cm width × 14 cm height. After the poplar seedlings were planted, 7 L of DI water was added to each pot. We ensured that the conditions were uniform in all the pots. After 20 days of recovery, the lateral bud of each poplar seedling was treated, so that only one lateral bud was selected for each seedling at the same position and treated with calcium.

### Experimental Design

The nutrient solution was formulated with deionized water according to Xie (2014). The pH of the solution was maintained at 5–6 by adding NaOH. In this culture solution, five different concentrations of Ca^2+^ (0, 2.5, 5, 10, and 20 mmol·L^−1^) were added and labeled as Ca0, Ca1, Ca2, Ca3, and Ca4, respectively (Paiva et al., 1998). Calcium was provided by anhydrous CaCl_2_, and other compounds that provided a large number of elements were KNO_3_, MgSO_4_·7H_2_O, KH_2_PO_4_, NaNO_3_, EATA-Na_2_, and FeSO_4_·7H_2_O. Trace elements were provided by H_3_BO_3_, MnCl_2_·4H_2_O, CuSO_4_·5H_2_O, ZnSO_4_·7H_2_O, and H_2_MoO_4_·H_2_O. After the seedlings were stable, the nutrient solution was poured every 5 days before the growth boom and once every 3 days during the growth period. During the growth of seedlings, each treatment was equipped with an air pump, which was continuously ventilated from 7:00 to 19:00 h and ventilated 1 h for every 2 h after 19:00 h. Other management measures were carried out in accordance with the routine procedures.

### Determination of the Growth Index of Poplar Seedlings

#### Growth of Poplar Seedlings

The heights and basal diameters of the seedlings were measured in July 2019 before destructive harvesting. The basal stem was measured with a Vernier caliper, accurate to 0.01 mm, and the plant height was measured with a ruler, accurate to 0.10 cm.

#### Biomass of Poplar Seedlings

During the harvest of seedlings, three non-destructive poplar seedlings were selected for each treatment in July 2019. The whole plant was removed from the planting pot. After washing, the whole plant was separated into roots, stems, and leaves with pruning shears and placed in an envelope for marking. These envelopes were then placed in an oven at 105°C for 30 min and dried to obtain a constant weight at 65°C. The dry weights of the roots, stems, leaves, and total plant biomass were determined using an analytical balance.

### Determination of Photosynthetic Characteristics of Poplar Seedlings

#### Photosynthetic Pigments

The photosynthetic pigments were extracted and determined by the ethanol method. The absorbance was measured at 665 nm and 649 nm after extraction with 95% ethanol for 48 h, and the chlorophyll-a and chlorophyll-b contents were measured. The following equations were used for calculations (Chen and Li, 2016):

Ca = 13.95A665–6.88A649

Cb = 24.96A649–7.32A665

where Ca and Cb are chlorophyll-a and chlorophyll-b, respectively, and A665 and A649 represent the absorbance values of photosynthetic pigment extracts at 665 and 649 nm, respectively.

#### Photosynthetic Parameters

Photosynthetic parameters were measured by using a photosynthetic instrument. During the peak growth of poplar seedlings (July 2019), sunny weather was chosen in the morning, three plants for each process were selected randomly, and the third functional or fully expanded leaf from the top of the plants was labeled with a red thread to investigate gas exchange. The gas exchange was measured using a Li-6400 photosynthesis system (Li-Cor Inc., Lincoln, USA) during 9:00–11:00 a.m. The photosynthetic photon flux (PPF) and CO_2_ concentration were maintained at 1,000 μmol·m^−2^·S^−1^ and 400 mol·mol^−1^, respectively. The net photosynthesis rate (Pn), stomatal conductance (Gs), transpiration rate (Tr), and intercellular CO_2_ concentration (Ci) were automatically recorded (Zhao et al., 2013; Fang et al., 2018).

#### Photosynthate

Soluble sugar concentrations were determined using the anthrone colorimetric method (Zhao, 2002). About 0.5 g of sample was placed in a centrifuge tube, and 10 ml of 80% ethanol was added. Next, the mixture was incubated at 95°C in a shaking water bath for 10 min and then centrifuged at 5,000 rpm for 10 min. The supernatants were collected, combined, and stored for later use. The extraction process was performed three times to ensure complete extraction of all the sugar content. Next, 5 ml of anthrone reagent was added to 0.2 ml of soluble sugar extraction liquid and placed in a water bath at 100°C for 10 min. The absorbance at 620 nm was measured to calculate the soluble sugar concentration according to the standard curve of glucose (Xie et al., 2018). Starch concentrations were determined using the perchlorate method (Wang et al., 1993). A total of 3 ml of H_2_O was added to the above sediment, and it was then placed in a 100°C water bath for 15 min. After cooling to room temperature, a total of 2 ml of 9.2 mol·L^−1^and 4.6 mol·L^−1^ of HClO_4_ and H_2_O were added to the above sediment. It was centrifuged at 4,000 rpm for 10 min. The supernatant was added to achieve a 50 ml constant volume for measuring the absorbance at 620 nm using the anthrone method. Then, the starch concentration was calculated according to the standard curve for glucose. Soluble sugar and starch concentrations were calculated on the basis of dry mass (Chen and Li, 2016).

#### Chlorophyll Fluorescence

The chlorophyll fluorescence parameters were determined in July 2019 at dusk. The OJIP curve for the leaves of poplar seedlings was prepared by using a portable, rapid chlorophyll fluorescence analyzer after leaf clamping and darkening treatment for 20 min.

### Determination of the Stress Tolerance of Poplar Seedlings

#### Water Use Efficiency

The iWUE value calculated from δ^13^C was used to characterize the water use efficiency of plants. The leaves were first crushed using a plant leaf drying crusher and then using a ball mill, and finally passed through a 100-mesh sieve. After complete drying, the sample weighed approximately 0.7 mg and was tightly wrapped in a tin boat. Then, δ^13^C was measured by a stable isotope mass spectrometer (DELTA V Advantage Isotope Ratio Mass Spectrometer), and the iWUE value was calculated using the following formula (Song et al., 2015; Ren et al., 2020):


$$\begin{array}{l} \begin{array}{cc} \text{iWUE }=\text{ A}/\text{Gs } & =\;\left(\text{Ca}-\text{Ci}\right)/1.6\\ \;=\text{ Ca }\left(1-\text{Ci}/\text{Ca}\right)/1.6\\ \;=\text{ Ca }\left(\text{b}-\text{Δ}13\text{C}\right)/1.6\left(\text{b}-\text{a}\right) \end{array} \end{array}$$

where A represents the net photosynthetic rate, Gs stands for stomatal conductance, and Ca and Ci are CO_2_ pressure values in the atmosphere and leaf cells, respectively. A and b represent the partial effects of CO_2_ diffusion into stomata and the partial effect of stomatal photosynthetic carboxylase RUBP on carbon isotopes, respectively.

#### Antioxidant Enzymes

For the determination of antioxidant enzymes, 0.4 g of samples was taken from fresh leaves, stored in a frozen pipe, fixed with liquid nitrogen, and stored in a refrigerator at −80°C. At the time of measurement, samples were taken according to the mark and put into a mortar. Then 5 ml of precooled phosphate buffer was added, the sample was ground, the homogenate was centrifuged at 13,000 rpm at 4°C for 15 min, and the supernatant was placed into a centrifuge tube for reserve (three repeats for each sample). Peroxidase (POD) level was determined by the guaiacol method. Catalase (CAT) level was determined based on ultraviolet absorption by hydrogen peroxide. Superoxide dismutase (SOD) level was determined by methionine (Perveen et al., 2020).

### Nutrient Elements in Each Organ

The total carbon and nitrogen contents were measured using a stable isotope mass spectrometer (DELTA V Advantage Isotope Ratio Mass Spectrometer). To measure total P content, samples were first digested with nitric acid and hydrogen peroxide, and then the extracted solutions were analyzed using an ultraviolet photometer (Shang et al., 2018; Zhang et al., 2018). The concentrations of potassium and calcium were measured using a flame atomic absorption spectrophotometer (Jing et al., 2018).

### Statistical Analysis

Excel and SPSS 22.0 software programs were used for sorting and drawing, statistical analysis, differential analysis, correlation analysis, and principal component analysis. All experiments were conducted with three replicates, and the results are expressed as the mean ± standard error (SE) values. The different letters in the chart indicate that the differences in each index between different calcium treatments reached the significance level of 5%.

## Results

### Growth of Poplar Seedlings

**Figure 1:**
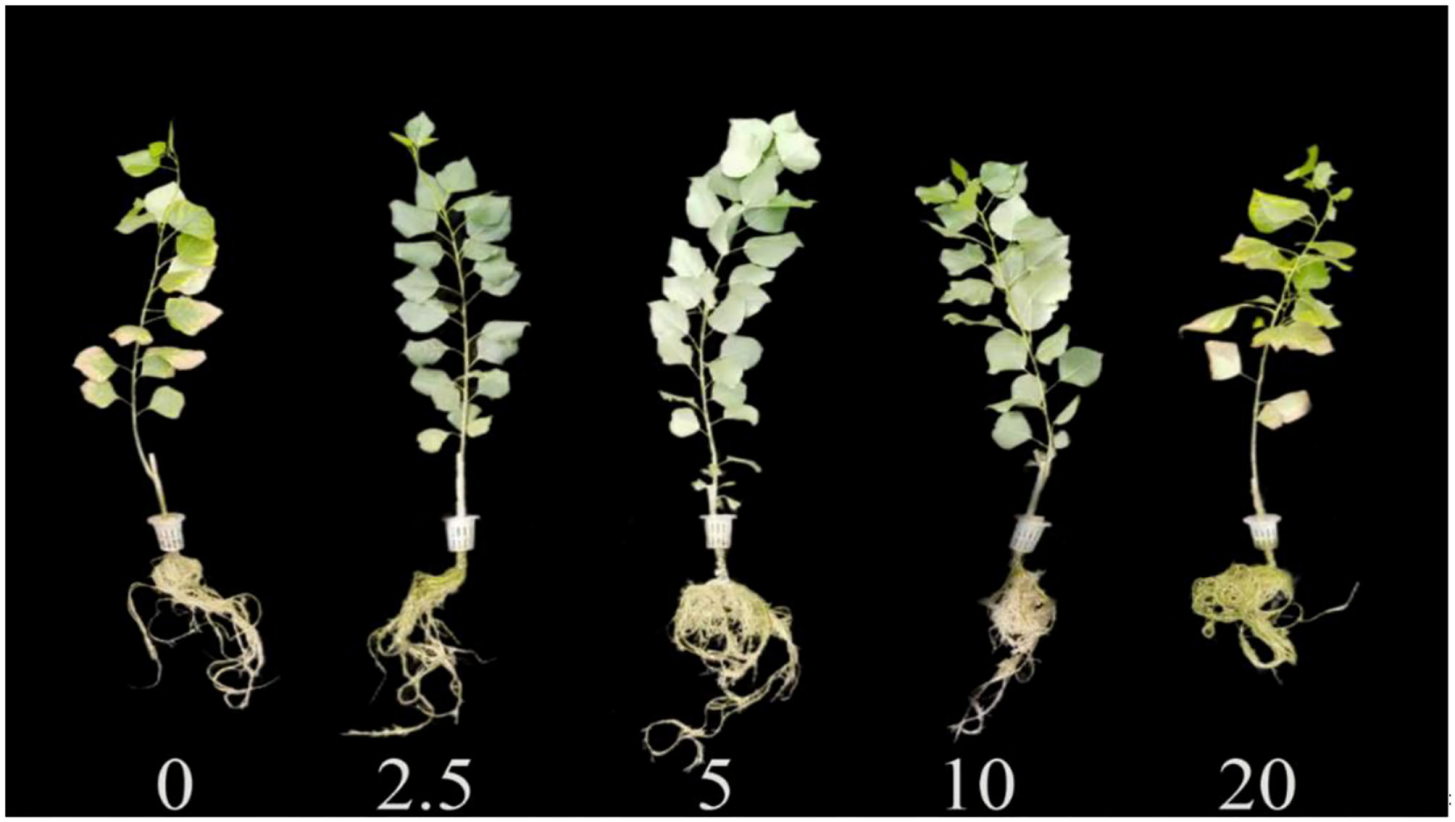
Growth of poplar seedlings exposed to different calcium concentrations.

**Figure 2:**
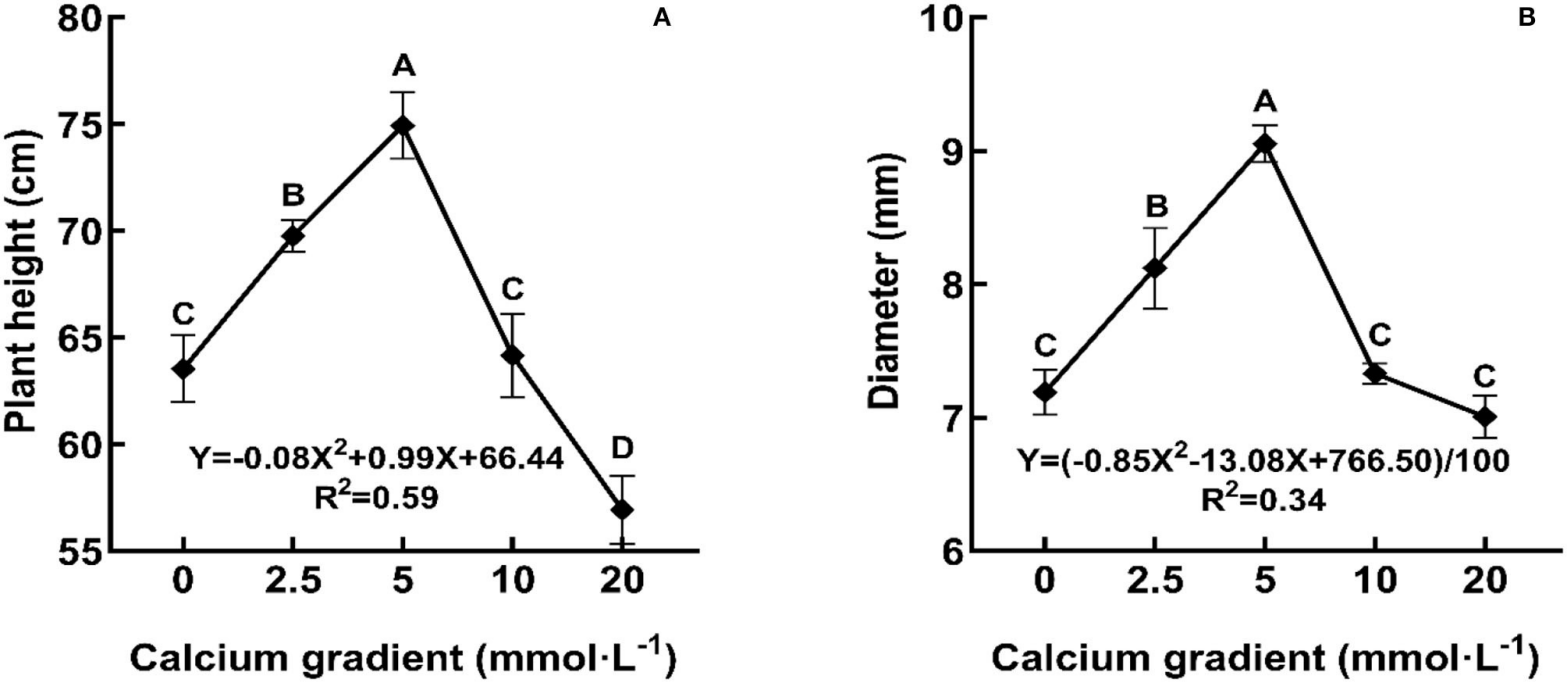
Growth of poplar seedlings in different treatments. Each column represented the mean ± SE values, *n* = 3; different capital letters indicate significant differences between treatments of calcium addition (*P* < 0.05).

In general, the indicators of growth and biomass showed an increasing trend initially and then decreased with increasing calcium concentrations (Figures 1, 2; Table 1). With the increasing gradient of calcium, the best value for all indicators was observed in the treatment with 5 mmol·L^−1^ of calcium, and there were significant differences with other treatments except for root biomass (*P* < 0.05). The maximum plant height and diameter increased by 17.94 and 25.91%, respectively, compared to plants with no calcium treatment (Figure 2). When Ca^2+^ concentration was 5 mmol·L^−1^, the root biomass, stem biomass, leaf biomass, and total biomass increased by 15.41, 24.21, 13.05, and 17.31%, respectively, compared to the plants with no calcium treatment (Table 1). However, when excess calcium was applied, there was no significant increase in the biomass of poplar seedlings compared to no calcium treatment.

**Table 1:** Biomass of poplar seedlings in different treatments.

**Calcium gradient (mmol·L^**−1**^)**	**Root biomass (g)**	**Stem biomass (g)**	**Leaf biomass (g)**	**Biomass (g)**
0	8.50 ± 0.124B	9.13 ± 0.239BC	10.96 ± 0.146B	28.59 ± 0.079C
2.5	9.40 ± 0.147A	10.04 ± 0.100B	10.83 ± 0.052B	30.27 ± 0.167B
5	9.81 ± 0.091A	11.34 ± 0.164A	12.39 ± 0.037A	33.54 ± 0.231A
10	8.06 ± 0.015BC	9.32 ± 0.106BC	11.08 ± 0.008B	28.60 ± 0.104C
20	7.66 ± 0.040C	8.71 ± 0.154C	8.78 ± 0.104C	25.15 ± 0.288D

*Each column and table show the mean ± SE value, n = 3. Different capital letters indicate a significant difference between treatments of calcium addition (P < 0.05)*.

### Photosynthetic Characteristics of Poplar Seedlings

**Figure 3:**
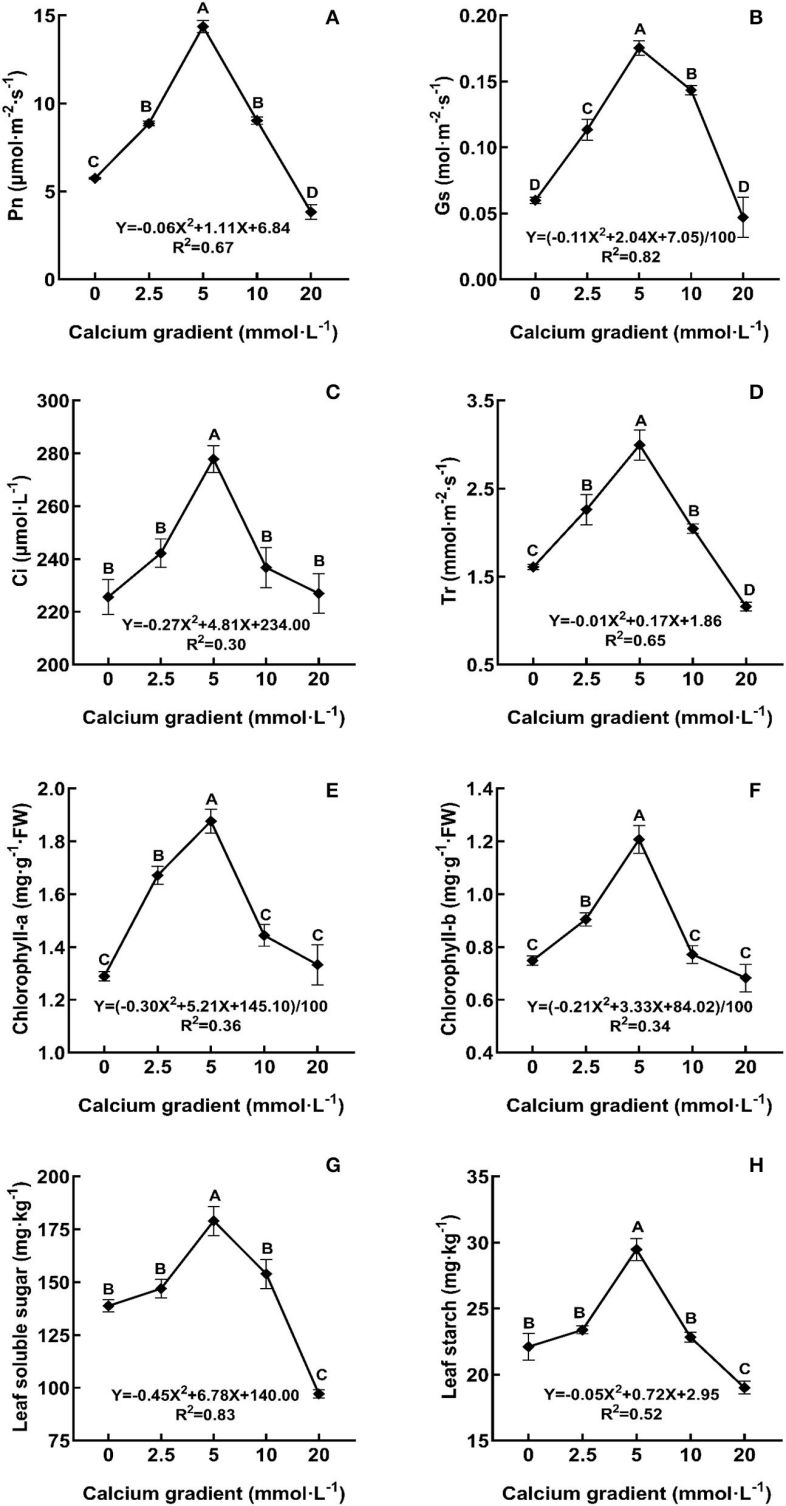
Photosynthetic characteristics of poplar seedlings in different treatments. Each column shows the mean ± SE value, *n* = 3. Different capital letters indicate significant differences between treatments of calcium addition (*P* < 0.05).

In general, the indicators of photosynthetic characteristics first showed an increasing trend and then a decreasing trend with increasing calcium concentration (Figure 3). With the increasing gradient of calcium addition, the best value for all photosynthetic indicators occurred in the treatments with 5 mmol·L^−1^ calcium, and there were significant differences with other treatments (*P* < 0.05). The Pn, Gs, Ci, and Tr levels increased by a maximum of 149.94, 191.67, 23.14, and 86.14%, respectively, compared to those in the absence of calcium treatment. A similar increasing trend was also observed for the indicators of chlorophyll-a, chlorophyll-b, leaf soluble sugar, and leaf starch levels, which increased by 45.54, 45.80, 29.00, and 33.31%, respectively. However, when excess calcium was applied, the photosynthetic capacity of poplar seedlings was weakened.

### Chlorophyll Fluorescence Parameters of Poplar Seedlings

**Figure 4:**
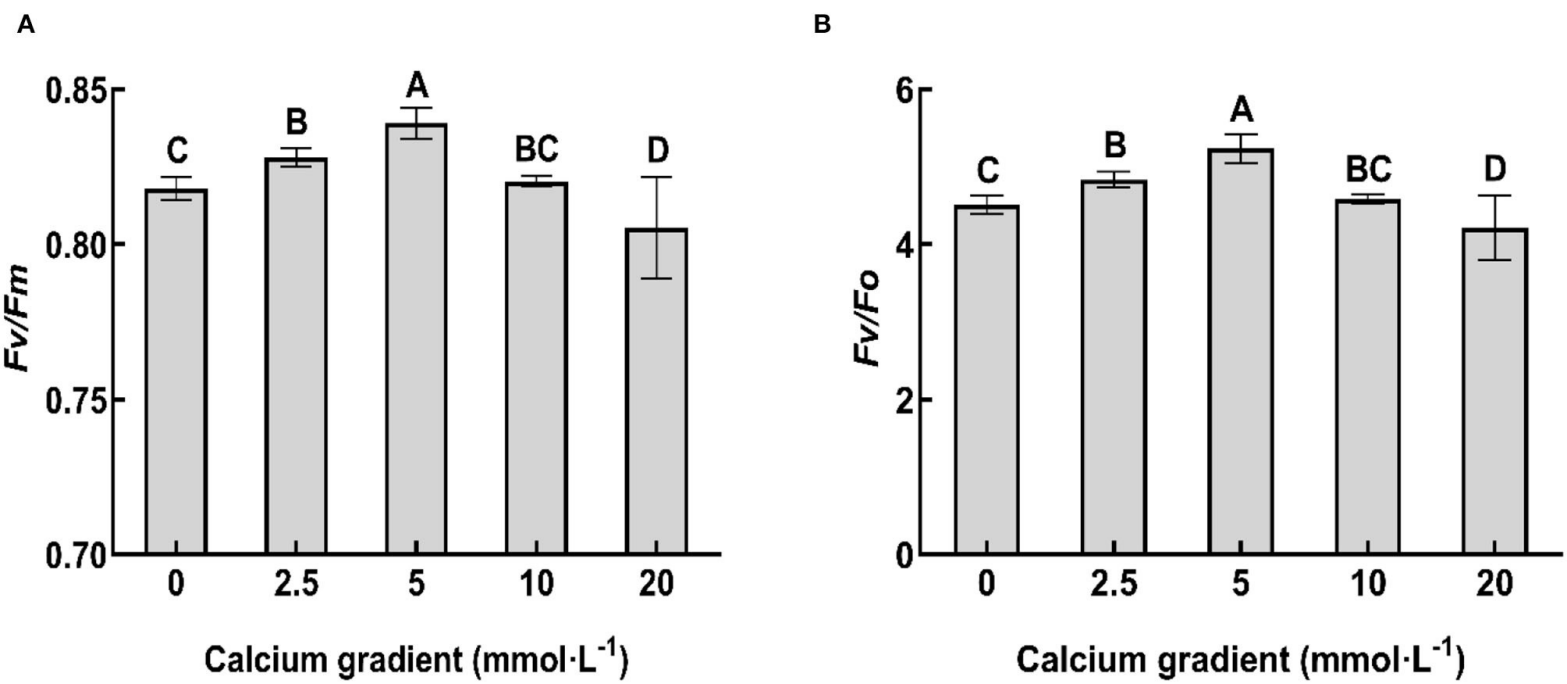
Chlorophyll fluorescence parameters of poplar seedlings in different treatments. *Fv/Fm* ratio indicates the maximum photochemical efficiency, and *Fv/F0* ratio indicates the potential photochemical efficiency. Each column represents the mean ± SE value, *n* = 3; different capital letters indicate significant differences between treatments with different concentrations of calcium (*P* < 0.05).

In general, the chlorophyll fluorescence parameters showed an increasing trend in the beginning and then decreased with increasing calcium concentration (Figure 4). With the increasing gradient of calcium addition, the best values for *Fv/Fm* and *Fv/F0* ratios occurred in the treatments with 5 mmol·L^−1^ calcium, and there were significant differences with other treatments (*P* < 0.05). The ratios increased by 2.57 and 16.01%, respectively, compared to no calcium treatment. However, when the calcium concentration was 20 mmol·L^−1^, the values of all the chlorophyll fluorescence parameters were lower than those obtained without calcium treatment (*P* < 0.05).

### Stress Tolerance of Poplar Seedlings

**Figure 5:**
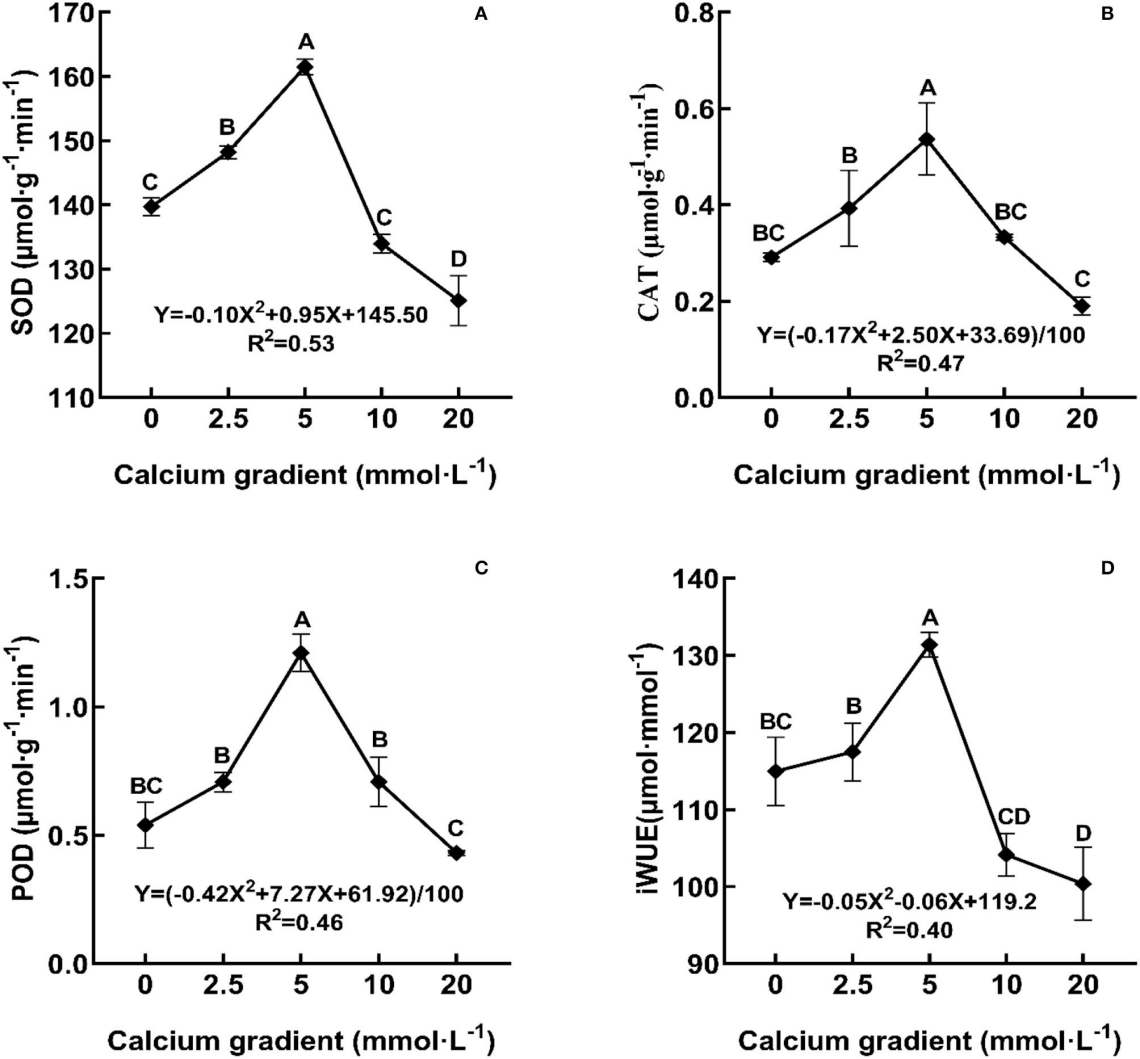
Effect of calcium on stress tolerance of poplar seedlings. POD, peroxidase; CAT, catalase; SOD, superoxide dismutase; iWUE, long-term water use efficiency. Each column represented the mean ± SE values, *n* = 3; different capital letters indicate significant differences between treatments of calcium addition (*P* < 0.05).

In general, the indicators of antioxidant enzyme activities and iWUE showed an increasing trend in the beginning and then decreased with increasing calcium concentration (Figure 5). With the increasing gradient of calcium addition, the best values for SOD, CAT, POD, and iWUE occurred in the treatments with 5 mmol·L^−1^ calcium, and there were significant differences with other treatments (*P* < 0.05). The parameters increased by 15.57, 84.19, 124.30, and 14.28%, respectively, when compared to no calcium treatment. However, when the calcium concentration was 20 mmol·L^−1^, the antioxidant enzyme activities and iWUE were lower than those obtained without calcium treatment.

### C, N, P, K, and Ca Absorption and Distribution of Poplar Seedlings

**Figure 6:**
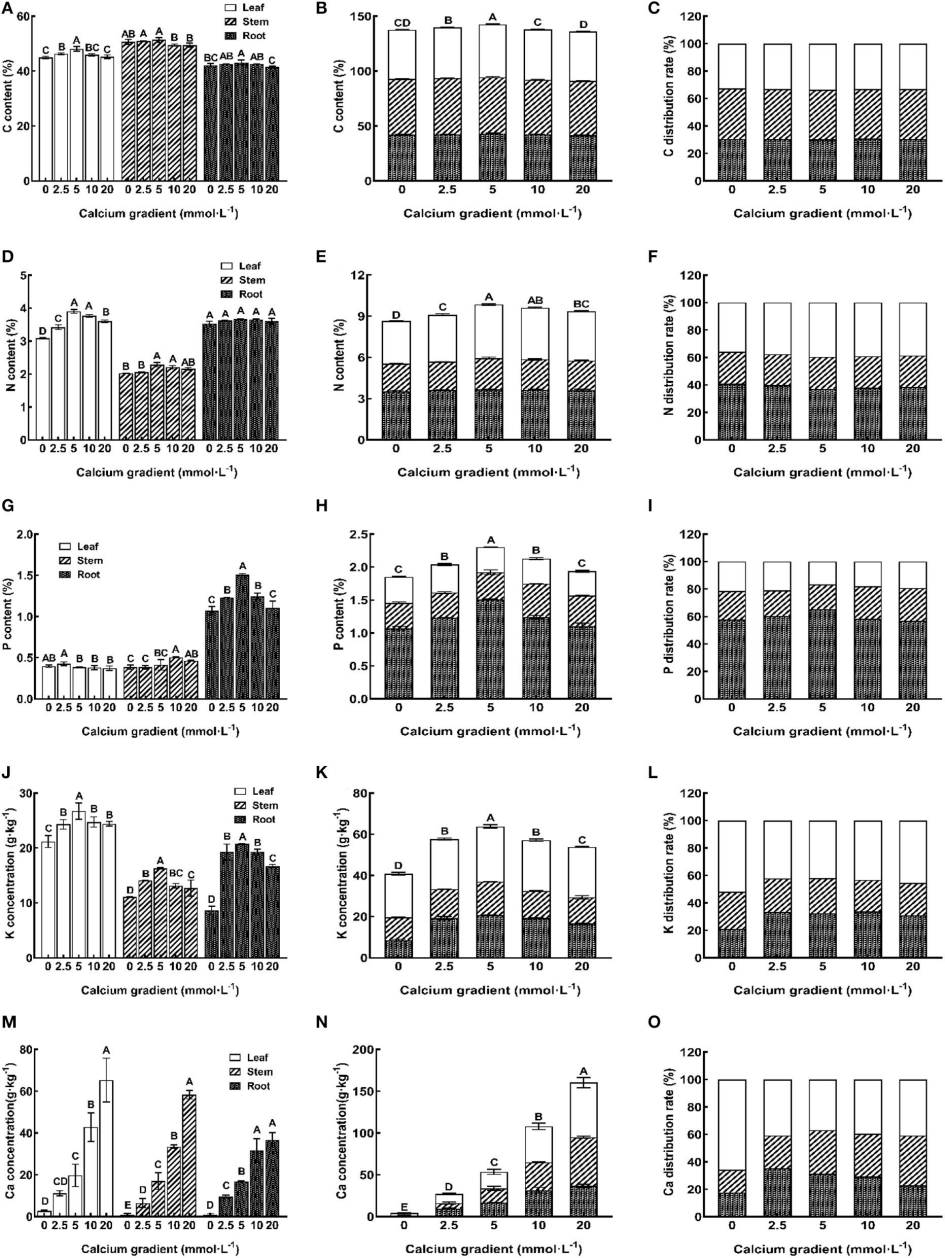
Effect of calcium on nutrient element absorption and distribution of poplar seedlings. Each column shows the mean ± SE value, *n* = 3. Different capital letters indicated significant differences between treatments of calcium addition (*P* < 0.05).

The results showed that endogenous concentrations of C, N, P, and K in the leaves, stems, roots, and the whole plant are influenced by different Ca supply levels and showed an increasing trend initially and then decreased (Figures 6A,B,D,E,G,H,J,K). The 5 mmol·L^−1^ calcium treatment resulted in the highest endogenous concentrations of C, N, P, and K, followed by 2.5 mmol·L^−1^ and 10 mmol·L^−1^ calcium treatments (except for the best P content of leaves and stems was observed with the 2.5 mmol·L^−1^ and 10 mmol·L^−1^ concentrations). There were significant differences in the concentrations of all other nutrient elements in all other organs with different calcium treatments, except for the N content of roots (*P* < 0.05). A different trend was observed for the Ca concentration in plant organs; the Ca accumulation rate in each tissue increased gradually with an increase in the doses of Ca ions (Figures 6M,N). Without Ca supply, the Ca concentration was significantly lower. The 10 mmol·L^−1^ and 20 mmol·L^−1^ Ca treatments led to a higher accumulation of Ca in the leaves, stems, and whole plant, and there are significant differences with other treatments except for roots (*P* < 0.05).

The percentage of nutrient elements in each organ in all the tissues reflects the distribution of nutrient elements in the trees and the regularity of the migration of nutrient elements in these tissues (Xing et al., 2021). The results showed that C, N, K, and Ca in the 5 mmol·L^−1^ Ca treatment group were mainly distributed in the leaves (Figures 6C,F,L,O), but P was mainly distributed in the roots (Figure 6I). With the increase in the concentration of Ca, the P, K, and Ca distribution rates of leaves first showed a decreasing trend and then an increasing trend, and roots showed an adverse trend. The N distribution rate of leaves first showed an increasing trend and then a decreasing trend, and roots showed a decreasing trend first and then an increasing trend, while there was no significant change in the distribution rate of Ca in leaves, stems, or roots. It is evident that an appropriate Ca supply could promote the distribution of N to the leaf system and P and K distribution to the root system, but since Ca is known to have a low transference capacity in plants, the Ca distribution rate was not significantly different between the different treatments, except for the 0 mmol·L^−1^ treatment.

### C:N:P Stoichiometry of Poplar Seedlings

**Figure 7:**
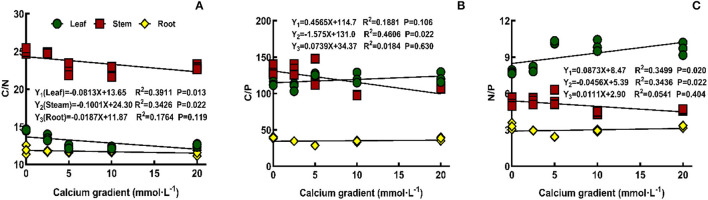
Effect of calcium on C/N, C/P, and N/P ratios of poplar seedlings.

The overall analysis results showed that (Figure 7) there was no correlation between the concentration of exogenous calcium and C/N, C/P, and N/P ratios of poplar roots or between the concentration of exogenous calcium and the C/P ratio of poplar leaves (*P* > 0.05). However, exogenous calcium concentration had a significant negative correlation with C/N (*P* < 0.05) and a significant positive correlation with N/P (*P* < 0.05) ratio in the leaves of poplar seedlings, and there was a significant negative correlation between exogenous calcium concentration and the C/N, C/P, and N/P ratios in stems (*P* < 0.05). With increasing exogenous calcium concentration, C/P in leaves gradually increased, while C/P in leaves and C/N, C/P, and N/P ratios in stems gradually decreased.

## Discussion

### Effect of Ca Supply Level on the Growth, Photosynthesis, and Stress Tolerance of Poplar Seedlings

Calcium is an essential nutrient for plant growth. Exogenous calcium can regulate the expression of plant growth genes, maintain cell function, promote plant growth and development, and improve plant tolerance to the environment (Naeem et al., 2017; Pathak et al., 2020). Previous studies showed that adding calcium fertilizer could significantly increase the biomass of Chinese cabbage, and when the calcium concentration was 600 mg·kg^−1^, the biomass of Chinese cabbage was the highest (Liu et al., 2009). For *Zoysia japonica*, an increase in the above- and below-ground fresh biomass was observed for all CaCl_2_ pretreatment groups, and the greatest increase was observed for the 10 mmol·L^−1^ CaCl_2_ pretreatment group (Xu et al., 2013). Foliar Ca^2+^ markedly improved maize growth, photosynthesis, stomatal conductance, transpiration rate, and accumulation of total soluble sugars (Naeem et al., 2018) and also improved the quality of sweet cherries (Correia et al., 2019). The foliar application of Ca^2+^ to sugar beet enhanced the plant biomass (Hosseini et al., 2019), and calcium fertilizer promoted the growth of poplar roots (Petrochenko et al., 2019). When plants are deficient in Ca, the cell wall cannot be formed, because the cell division and formation are affected. Lysine degradation is affected, leading to the accumulation of pipecolic acid at the onset of this metabolic pathway and resulting in a decrease in the accumulation of plant biomass and the possible development of physiological diseases (Cinčerová, 1976; De Freitas et al., 2016). However, excessive Ca not only results in cellular toxicity and destroys normal biochemical metabolism, nutrient element metabolism, and other processes in plants, but also has an impact on plant morphology and alterations in the internal structure, such as overly rigid cell walls (Tyler and Olsson, 2009; Conn et al., 2011; Cybulska et al., 2011). For example, Ca deficiency or Ca overload in tomato plants reduces leaf size and plant biomass (Hao and Papadopoulos, 2004). The present study also showed similar results. When exogenous calcium was applied to poplar seedlings, the biomass of each organ and the total biomass of poplar seedlings first increased and then decreased, reaching a maximum when the calcium gradient was 5 mmol·L^−1^. Therefore, the amount of calcium can influence plant growth and biomass accumulation. The application of exogenous calcium led to distinct changes in the levels of nutrients in leaves, such as Mg, N, P, etc., which further led to an increase in the photosynthetic pigment and photosynthetic product levels and eventually enhanced plant growth and biomass.

Photosynthesis is the process by which green plants absorb light energy, assimilate carbon dioxide and water, make raw and processed materials, and release oxygen (Okafor and Okeke, 2017). It is the basis for dry matter accumulation in plants. Photosynthesis is affected by interactions with various environmental factors, such as light, temperature, water, and minerals (Ke, 2001; Feller and Vaseva, 2014; Rubenovna and Ramazanovich, 2018). Chlorophyll is an important photosynthetic pigment that absorbs and transforms light energy (Kumara et al., 2021). Its content is closely related to thephotosynthetic capacity of plants (Mohsenpour and Willoughby, 2013). Chlorophyll fluorescence kinetics can be used to monitor multiple photosynthetic reactions and reflect the success of plants. Changes in chlorophyll fluorescence are closely related to the absorption, transfer, dissipation, and distribution of light energy. By measuring changes in chlorophyll fluorescence parameters, the adaptability of plants to stressful environments can be well identified (Peng et al., 2021; Zheng et al., 2021). When *Fv/Fm* ratio is less than 0.8, it indicates that plants are under stress and their growth and development are severely inhibited. Calcium is involved in the process of electron transport and phosphorylation in plant photosynthesis, which directly affects the plant photosynthetic mechanism (Ferguson and Drobak, 1988). Several studies have established an essential role of Ca in maintaining photosynthesis by modulating gas exchange in leaves, PSII processes, carbohydrate metabolism, and expression of chlorophyll synthesis-related genes (Zhang et al., 2019). For example, the stress related to calcium deficiency reduces the chlorophyll content of lettuce (Fan and Yin, 2002), and the net photosynthetic rate, stomatal conductance, and other parameters of potato plants showed a downward trend (Xin, 2008). An appropriate amount of exogenous calcium enhanced the net photosynthetic rate, stomatal conductance, intercellular CO_2_ concentration, maximum quantum efficiency of photosystem II photochemistry, actual photochemical efficiency of PSII, photochemical quenching coefficient, and non-photochemical quenching coefficient (He et al., 2018). When the calcium concentration was more, the plant height, biomass, net photosynthetic rate, and chlorophyll content of tomato plants decreased significantly (Zhou et al., 2012). Additionally, a transient increase in Ca causes short-term stomatal closure; therefore, a high Ca concentration does not seem to be directly related to transpiration (Xing et al., 2021). In addition, other studies have found that calcium can affect leaf bud differentiation. When the calcium concentration was 0, the number of differentiated buds decreased, and the greatest number was achieved at 2 MS, but growth was inhibited at 4 MS, at which concentration the number of green leaves was moderate (Li and Liu, 2007). In our study, we found that the photosynthetic characteristics of poplar seedlings were significantly affected by the addition of calcium, and the chlorophyll content, photosynthetic parameters, and chlorophyll fluorescence parameters increased first and then decreased with increasing calcium concentration. This is similar to previous research results. Therefore, calcium at an optimal level is critical for plant growth. It can affect the gas exchange process related to photosynthesis by regulating stomatal movement (Wang et al., 2019). Moreover, a proper amount of calcium ions may improve the integrity and stability of chloroplast structure, and enhance the activities of Rubisco and PEP carboxylase enzymes, thus improving the carboxylation efficiency of carbon dioxide and the activity of ATPase on the membrane and thereby the photosynthetic level of plants.

The parameters like POD, CAT, SOD, and iWUE were measured to identify the resistance capacity of the plant to stress conditions. iWUE reflects the energy conversion efficiency in plant production, is an index used to measure the relationship between crop yield and water consumption, and is also one of the comprehensive indices used to evaluate plant growth suitability under water deficit. In current forest ecology, the water use efficiency (iWUE) of trees is very important in the study of water resources and for the restoration of forest vegetation (Xi et al., 2018). The antioxidant enzyme system can scavenge free radicals in the cell and protect the cell membrane system from damage. Some of the antioxidant enzymes often found in plants are POD, SOD, and CAT (Guo et al., 2018). Previous studies on *Allium sativum* and *Pinus massoniana* seedlings showed the effects of elevated calcium concentration on the expression levels of POD, SOD, and CAT. In *Allium sativum*, these antioxidant enzymes first increased and then decreased. In contrast, in *Pinus massoniana* needles, these antioxidant enzymes first decreased and then increased at different growth stages (Li et al., 2013; Li and Zhou, 2017). In addition, under some stress factors, such as cadmium stress, suitable exogenous calcium can improve the activities of SOD, POD, and CAT in plant seedlings (Siddiqui et al., 2012), and optimal Ca supplementation upregulated the activities of the assayed antioxidant enzymes and the contents of non-enzymatic antioxidants (ascorbate, glutathione, and tocopherol), thereby reflecting the amelioration of NaCl-induced oxidative damage (Elkelish et al., 2019). Calcium is closely related to plant water use efficiency; for example, a suitable concentration of calcium fertilizer can increase iWUE, fresh yield, and fruit quality of tomato (Hong-Bo et al., 2008; Yang et al., 2012). The results showed that the iWUE and antioxidant enzyme activity of poplar seedlings first showed an increasing trend and then a decreasing trend, and reached maximum values when the calcium concentration was approximately 5 mmol·L^−1^. At this concentration, the stress resistance of poplar seedlings was the best.

In conclusion, for poplar seedlings, when the concentration of exogenous calcium was 5 mmol·L^−1^, the photosynthetic capacity, stress resistance, and growth effect were maximum.

### Effect of Ca Supply Level on the Nutrient Absorption, Distribution, and Utilization, and Stoichiometry of Poplar Seedlings

Nutrient elements play an important role in plant growth and development by affecting plant physiological processes, such as stomatal conductance, photosynthetic rate, and water use efficiency, which can directly or indirectly affect plant growth (Ma, 2017). In higher plants, interactions between nutrients occur when the supply of one nutrient affects the absorption, distribution, or function of another nutrient. Interactions between nutrients can be assessed by examining both the relationship between nutrient supply and growth and the relationship between nutrient concentrations in plants and growth (Robson and Pitman, 1983; Sevanto et al., 2020). Currently, a large number of studies on the C, N, and P ecological stoichiometry changes in plants focus on global changes, such as elevated ozone levels (Shang et al., 2018), plant management strategies (Heyburn et al., 2017; Chen and Chen, 2021), N deposition (Huang et al., 2018), elevated CO_2_ concentrations (Du et al., 2018), and their interactions. However, few studies have been conducted that determined the effect of calcium on C, N, and P ecological stoichiometry, and most studies that evaluated the effect of calcium on plant growth are mainly focused on cash crops, such as pepper (*Capsicum annuum*) (Akladious and Mohamed, 2018), rice (Liang et al., 2021a), and tomato (Guo et al., 2018). Therefore, it is of great significance to explore the relationship between nutrient stoichiometry characteristics and the growth of poplar seedlings to reveal suitable vigor theory and select growth evaluation indices.

This study showed that N, P, K, and C levels in each organ of poplar seedlings first increased and then decreased with increasing application of exogenous calcium content, and their maximum levels were reached when the concentration of exogenous calcium was 5 mmol·L^−1^. In contrast, calcium levels in each organ of poplar seedlings increased continuously with increasing exogenous calcium content. These results are similar to those of previous studies. For example, the addition of calcium sulfate to nutrient solution significantly increased leaf K^+^, Ca^2+^, and N levels in tomatoes (Tuna et al., 2007). Also, exogenous Ca^2+^ was shown to promote the absorption of K^+^ in poplar roots after prolonged exposure to salinity and to retain K^+^/ Na^+^ homeostasis in roots (Sun et al., 2009). Another study showed that foliar application of Ca^2+^ improved the photosynthetic capacity of leaves, thus increasing the accumulation of C in plants and enhancing dry matter production (Song et al., 2020). After calcium treatment, the content of calcium and phosphorus increased significantly (Niu et al., 2018). The reasons for these findings may be as follows: An appropriate amount of Ca^2+^ can maintain the integrity of the plant cell membrane and ensure the selective permeability of the cell membrane, and when the concentration of Ca^2+^ is appropriate, it can exhibit a synergistic effect with other ions (such as N) to promote the absorption of other nutrient elements. In addition, with the increase in exogenous calcium content, the proportion of C in roots, stems, and leaves did not change, while the proportion of N in leaves first increased and then decreased, and the proportion of other elements, such as P, K, and Ca, in leaves first decreased and then increased.

In this study, the calcium content of poplar was found to be positively correlated with the N/P ratio in leaves and negatively correlated with the N/P ratio in stems. However, N/P <14 indicates that plant growth in this study was mainly restricted by N content. At the same time, the C/N ratio in the leaves and stems of poplar and the C/P ratio in stems are significantly negatively correlated with exogenous calcium content, indicating that leaves need more N and stems need more N and P to fix C under the conditions of calcium deficiency and that the NUE and PUE were low. No correlation was found between exogenous calcium or C/P in leaves and stoichiometry in roots.

## Conclusion

Spatial heterogeneity of calcium affects the growth of poplar seedlings in different regions. There is an optimal calcium gradient that is most suitable for the growth of poplar seedlings. The effects of calcium gradient were determined by examining photosynthetic characteristics, water use efficiency, and antioxidant enzyme activity, and the results showed that 5 mmol·L^−1^ calcium concentration is the most suitable condition for the growth of poplar seedlings. Different nutrient elements have different absorption centers. Exogenous calcium could promote the absorption of nutrient elements and regulate the transfer of nutrients between organs in plants. This could change the stoichiometric characteristics of poplar seedlings; for example, the N/P ratio in leaves of poplar seedlings could be increased, thus improving photosynthesis and dry matter accumulation of poplar seedlings, enhancing stress resistance, and promoting growth. Once the calcium concentration was over 10 mmol·L^−1^, the physiological indices of the poplar seedlings were significantly reduced, showing that adverse effects of calcium stress occurred due to excessive amounts of exogenous calcium. Therefore, there is an optimal calcium concentration for the growth of poplar seedlings. The results of this study provide a theoretical basis for the later studies on poplar plantations.

## Data Availability Statement

The original contributions presented in the study are included in the article/supplementary material, further inquiries can be directed to the corresponding author/s.

## Author Contributions

YZ and HL proposed the idea. XW and CR performed experiments. XW process and analyze data, production of figures, and write this paper. All authors participated in the experiments and reviewed the manuscript. All authors contributed to the article and approved the submitted version.

## Funding

The work was supported by the National Natural Science Foundation of China (31700552, 41450007, 31800364, and 31400611) and the Doctoral Research Start-up Fund (880416020).

## Conflict of Interest

The authors declare that the research was conducted in the absence of any commercial or financial relationships that could be construed as a potential conflictof interest.

## Publisher's Note

All claims expressed in this article are solely those of the authors and do not necessarily represent those of their affiliated organizations, or those of the publisher, the editors and the reviewers. Any product that may be evaluated in this article, or claim that may be made by its manufacturer, is not guaranteed or endorsed by the publisher.
